# Prognostic role of oxytocin receptor in colon adenocarcinoma

**DOI:** 10.1515/med-2021-0387

**Published:** 2021-11-22

**Authors:** Junjie Sun, Zhenyu Xu, Yong Mao, Ting Zhang, Yan Qin, Dong Hua

**Affiliations:** Department of Oncology, The Second Affiliated Hospital of Soochow University, Suzhou, 215004, Jiangsu, P. R. China; Department of Oncology, The Affiliated Hospital of Jiangnan University, No. 200, Huihe Road, Wuxi, 214000, Jiangsu, P. R. China; Department of Pathology, The Affiliated Hospital of Jiangnan University, No. 200, Huihe Road, Wuxi, 214000, Jiangsu, P. R. China; Department of Oncology, The Affiliated Wuxi People’s Hospital of Nanjing Medical University, No. 299, Qingyang Road, Wuxi, 214000, Jiangsu, P. R. China

**Keywords:** oxytocin receptor, colorectal cancer, The Cancer Genome Atlas, poor prognosis, immune infiltration

## Abstract

The oxytocin receptor (OXTR) is directly involved in the pathological mechanisms of multiple cancers, including breast cancer, prostate cancer, and ovarian cancer; however, the role of OXTR in the modulation of colon adenocarcinoma (COAD) growth, metastasis, and clinical prognosis remains to be elucidated. This study used systematic bioinformatics analysis to explore the effects of OXTR on modulating COAD growth and prognosis in patients with COAD. Compared with normal tissues, OXTR mRNA level was higher in COAD tissues, which was associated with tumor progression. Elevated mRNA level of OXTR also indicated a poor prognosis in COAD patients. Furthermore, high mRNA level of OXTR was significantly associated with pathways involved in cell cycle regulation and signal transduction pathways, including the hedgehog, mTOR, TGF-β, and Wnt signaling pathways. OXTR expression was significantly correlated with the infiltration level of type 2T helper cell, central memory CD8 T cell, CD56 bright natural killer cell, activated CD8 T cell, activated B cell, and Type 1T helper cell. Moreover, silencing OXTR inhibited cell proliferation, migration, and invasion, and arrested the cell cycle. In conclusion, high mRNA level of OXTR indicates poor prognosis.

## 1 Introduction

Colon adenocarcinoma (COAD), widely accepted as an illness that starts in the rectum or colon, ranks as the third most common cancer and the fourth leading cause of cancer-related death in the world [1,2,3]. Metastasis is usually present in most COAD patients at the first diagnosis, and this trend has been more severe over the last two decades due to changes in eating habits and living conditions [4]. Currently, the selective treatment for COAD is based on two common methods, surgery and chemotherapy, which are mainly administered based on the tumor stage [3]. The stage of COAD is mainly described by the TNM (tumor-node-metastasis) system initiated by the American Joint Committee on Cancer (AJCC) and the Union for International Cancer Control (UICC) [5]. Due to environmental pollution, poor dietary patterns, obesity, and dysbiosis of gut microbiota, the incidence of COAD, especially among the younger population, has largely increased in recent decades, which adds a heavy burden to the health care system and our society [6,7]. Thus, understanding the pathological mechanism for COAD is very urgent.

The processes of cell proliferation and related metastasis in COAD patients are very complex and mainly include the activation of oncogenes and the loss of function of tumor suppressor genes, leading to overactivation of signal transduction networks, including the TGF-β, Wnt/β-catenin, Smad, Notch, Hippo-YAP, MAPK, HIF-1, mTOR, and PI3K/Akt pathways, to promote cancer cell proliferation and metastasis [8,9,10,11,12,13,14,15,16].

The oxytocin receptor (OXTR) is a molecule that is responsible for the recognition of the hormone and neurotransmitter oxytocin. Previous studies show some evidence that OXTR-mediated signaling is implicated in multiple biological and pathological processes ranging from the involvement of OXTR in reproductive and social behavior to its promotion of multiple cancers [17,18,19]. The OXTR-oxytocin axis has been implicated in preventing the emergence of breast cancer [20]. Coupling of OXTR with Gi proteins enables the recognition of oxytocin to induce migration and metastasis in prostate cancer [21]. However, the role of the OXTR signaling network in COAD remains to be elucidated. Thus, this study aims to explore the role of OXTR in modulating the proliferation, metastasis, and prognosis of COAD patients by using bioinformatics analysis, which will be helpful for the diagnosis and treatment of COAD.

## 2 Materials and methods

### 2.1 Analysis of OXTR expression in various tumor tissues in The Cancer Genome Atlas (TCGA)

The expression levels of OXTR in various tumor tissues and adjacent normal tissues were analyzed in Tumor Immune Estimation Resource (TIMER, https://cistrome.shinyapps.io/timer), and the relationships between OXTR expression levels in various tumors and overall survival (OS) were analyzed in GEPIA2 (http://gepia2.cancer-pku.cn) [22]. The cutoff of the high OXTR group was 75%, and the cutoff of the low OXTR group was 25%.

### 2.2 Evaluation of OXTR expression at transcriptional level in COAD patients

RNA-sequence data of COAD patients were downloaded from TCGA and Gene Expression Omnibus (GEO: GSE9348, GSE32323, GSE38026, GSE44076, and GSE115313) database. After converting the “count” value from TCGA into logarithm, GraphPad Prism 8 was used to analyze the mRNA level of OXTR in COAD tissue and non-tumor tissue. Normalized values downloaded from GEO were used to analyze the mRNA levels of OXTR in COAD tissues and non-tumor tissues using GraphPad Prism 8.

### 2.3 OS rate assessment by Kaplan–Meier (KM) curves

According to the median expression value of OXTR at the mRNA level, COAD patients were divided into two groups, the high OXTR mRNA group and the low OXTR mRNA group. The OS in COAD patients was assessed by KM curves. Patients who were alive or disease-free for ≥5 years were evaluated as OS good. Patients who died of the disease or relapsed within 2 years were evaluated as OS poor.

### 2.4 ROC curve analysis

We used SPSS software (version 21.0) to draw the ROC curve and calculated the area under the curve (AUC) value to evaluate the ability of OXTR to identify COAD patients.

### 2.5 Gene set enrichment analysis

The RNA-sequence data of COAD patients from TCGA were converted into logarithms, the patients were divided into high expression groups and low expression groups according to the median value of OXTR, and then GSEA 3.0 software was used for GSEA analysis, including Gene Ontology (GO) and Kyoto Encyclopedia of Genes and Genomes (KEGG) enrichment analyses [23,24]. Pearson’s correlation analysis was used for ranking genes.

### 2.6 Construction of protein–protein interaction (PPI) and gene co-expression networks

We used the cBioPortal database to analyze the genes co-expressed with OXTR. Genes with Spearman correlation coefficient with OXTR expression >0.3 or <−0.3 were uploaded to Cytoscape software (version 3.7.1) to map the gene co-expression network.

We screened genes whose absolute value of Spearman correlation coefficient with OXTR expression was greater than 0.3 and uploaded them to the STRING website to analyze PPI, as described previously [25]. The threshold value is *P* value < 0.05. We removed protein nodes that do not interact with other proteins and input PPI pairs into Cytoscape software (Version 3.7.1) to build PPI network, and the top ten hub genes were identified according to the Cytoscape plug-in (degrees ranking of cytoHubba).

### 2.7 Analysis of immune infiltration

Gene expression RNA-sequence (TOIL RSEM fpkm, *n* = 10,535) and phenotype (Curated clinical data, *n* = 12,591) of TCGA Pan-Cancer (PANCAN) were downloaded from Xena (http://xena.ucsc.edu) [26]. The gencode.v36.annotation.gtf file was downloaded from GENCODE (https://www.gencodegenes.org) to convert TCGA Pan-Cancer expression TPM from ensembl gene id to Symbol, and merge the expression and survival data. According to the data of marker genes of immune cells described by Charoentong et al. [27], the GSVA package (version 1.36.3) in R software (version 4.0.2) was used to perform single-sample gene set enrichment analysis to identify immune infiltration. Finally, the relationship between the immune infiltration and the level of OXTR in TCGA-COAD (*n* = 329) samples was demonstrated.

### 2.8 Cell culture

All cell lines used in this study were obtained from Xiamen Immocell Biotechnology Co., Ltd (Xiamen, China). The human COAD cell lines HCT-8 (catalog number: IM-H099) and RKO (catalog number: IM-H409) were cultured in RPMI-1640 Medium (ATCC, Catalog No. 30-2001) with 10% horse serum and Eagle’s Minimum Essential Medium with 10% fetal bovine serum (FBS), respectively, and SW620 (catalog number: IM-H112) and SW480 (catalog number: IM-H111) were cultured in Leibovitz’s L-15 Medium (ATCC, Catalog No. 30-2008) with 10% FBS. Human normal colon epithelial cell line NCM-460 was cultured in DMEM (Gibco, Detroit, MI, USA) with 10% FBS.

### 2.9 Quantitative PCR (qPCR)

Cells were lysed with an RNA isolation kit (Sigma, catalog number: 83913-1EA) to extract total RNA which was reverse transcribed to cDNA using a HiScript II 1st Strand cDNA Synthesis kit (VAZYME, catalog number: R101-01/02, Nanjing, Jiangsu, China) according to the manufacturer’s instructions. qPCR analysis was performed using the obtained cDNA, the iQ5 Real-Time PCR Detection System (Bio-Rad Laboratories, Inc.), and a ChamQ SYBR qPCR Master Mix kit (Vazyme Biotech Co., Ltd). The relative expression levels of genes were normalized to the 18S rRNA levels using the 2^−ΔΔCq^ method. The primers used for qPCR are shown as follows. 18S forward primer: 5′-CGACGACCCATTCGAACGTCT-3′; 18S reverse primer: 5′-CTCTCCGGAATCGAACCCTGA-3′; OXTR forward primer: 5′-TCATCGTGTGCTGGACGCCTTT-3′; OXTR reverse primer: 5′-CGTGAACAGCATGTAGATCCAGG-3′. The data are expressed as the mean value ± standard deviation (SD) of three independent experiments.

### 2.10 Western blotting

Cells were lysed in RIPA buffer (Beyotime, catalog number: P0013C) to extract protein which was quantitated using a BCA protein concentration determination kit (Beyotime, catalog number: P0012S). 20 µg of protein was separated by 10% SDS-PAGE. Proteins were transferred to polyvinylidene fluoride membranes (Millipore, catalog number: IPVH00010), which were blocked with 5% skim milk and then incubated with OXTR antibody (23045-1-AP, 1:1,000, Proteintech, Wuhan, China) or GAPDH antibody (10494-1-AP, 1:20,000, Proteintech, Wuhan, China) for 2 h at 25°C, followed by incubation with HRP-conjugated goat anti-rabbit IgG (SA00001-2, 1:10,000, Proteintech, Wuhan, China) for 1 h at 25°C. The membranes were visualized using a typically enhanced chemiluminescent kit (Thermo Fisher Scientific). ImageJ v1.48 (National Institutes of Health) was used for densitometry. Three independent experiments were performed.

### 2.11 MTT assay

After seeded into 96-well plates with 1.0 × 10^4^ cells per well, the cells were transfected with small interfering RNA (siRNA) of OXTR (siOXTR) or the negative control of siOXTR (siNC). After 24, 48, or 72 h, 20 µL of MTT (5 mg/mL) per well was added and the cells were incubated at 37°C for 4 h. After the culture supernatant was carefully aspirated, 150 µL of DMSO per well was added, and the cell culture plate was shaken for 10 min to dissolve the crystals. Subsequently, the light absorption value of each well was measured at 490 nm on an enzyme-linked immunosorbent detector. The cell growth curve was plotted with time as the abscissa and absorbance as the ordinate. The data are represented in terms of mean value ± SD for sextuple wells.

### 2.12 Cell cycle assay

Cells seeded at 1.2 × 10^6^ cells per well were transfected with siRNA as described above. After 24 h, the cells were harvested and fixed in 70% ethanol at 4°C overnight. After permeabilized by 0.2% Triton X-100 containing 10 µg/mL RNase at 37°C, the cells were stained with propidium iodide (PI, 20 µg/mL) and analyzed using a flow cytometer. The experiments were performed thrice independently.

### 2.13 Transwell assay

Migration and invasion were measured without and with Transwell plates, respectively. A total of 1 × 10^5^ A-498 cells transfected with siOXTR or siNC in serum-free medium were plated in the upper chambers of the Transwell plates (8-µm pore size; Corning, Inc., Corning, NY, USA), and 10% FBS medium was added to the lower chambers of the Transwell plates. After incubation for 24 h at 37°C, the migrated and invasive cells were stained with 0.5% crystal violet. Stained cells were counted in six randomly-selected fields. The experiments were performed thrice independently.

### 2.14 Statistical analysis

All assays were performed independently at least 3 times. Statistical analysis of experimental data was performed using SPSS software 22.0 (IBM Corp.). Mann–Whitney test was performed for non-parametric data between two groups. Student’s *t* test (unpaired) for nonparametric and parametric data between two groups, one-way ANOVA followed by Tukey’s *post-hoc* test were used to identify the significant differences among multiple groups. Log-rank test was used for KM survival analysis. *p* < 0.05 was considered to indicate a statistically significant difference.

## 3 Results

### 3.1 The expression pattern of OXTR in various tumor tissues and its relationship with patients’ OS were analyzed

**Figure 1:**
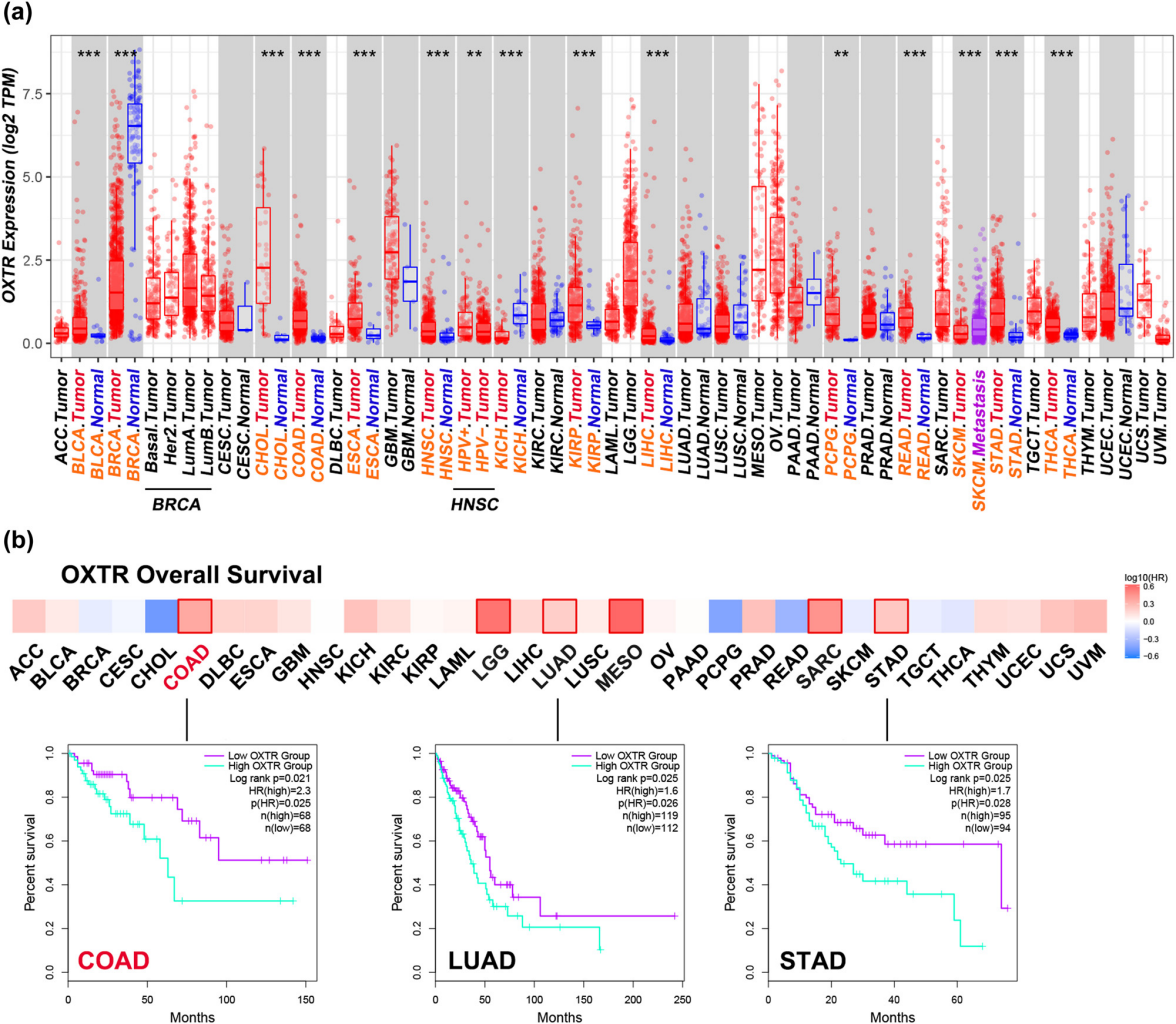
Analysis of OXTR expression in various tumor tissues in TCGA. (a) Transcriptional levels of OXTR in different tumor tissues and adjacent normal tissues. (b) The relationships between OXTR expression levels in various tumors and OS were analyzed by KM curve. COAD: low OXTR group: *n* = 68, high OXTR group: *n* = 68; LUAD: low OXTR group: *n* = 112, high OXTR group: *n* = 119; STAD: low OXTR group: *n* = 94, high OXTR group: *n* = 95. ACC: adrenocortical carcinoma; BLCA: bladder urothelial carcinoma; BRCA: breast invasive carcinoma; CESC: cervical squamous cell carcinoma and endocervical adenocarcinoma; CHOL: cholangiocarcinoma; COAD: colon adenocarcinoma; DLBC: lymphoid neoplasm diffuse large B-cell lymphoma; ESCA: esophageal carcinoma; GBM: glioblastoma multiforme; HNSC: head and neck squamous cell carcinoma; HPV: human papillomavirus; KICH: kidney chromophobe; KIRC: kidney renal clear cell carcinoma; KIRP: kidney renal papillary cell carcinoma; LAML: acute myeloid leukemia; LGG: brain lower grade glioma; LIHC: liver hepatocellular carcinoma; LUAD: lung adenocarcinoma; LUSC: lung squamous cell carcinoma; MESO: mesothelioma; OV: ovarian serous cystadenocarcinoma; PAAD: pancreatic adenocarcinoma; PCPG: pheochromocytoma and paraganglioma; PRAD: prostate adenocarcinoma; READ: rectum adenocarcinoma; SARC: sarcoma; SKCM: skin cutaneous melanoma; STAD: stomach adenocarcinoma; TGCT: testicular germ cell tumors; THCA: thyroid carcinoma; THYM: thymoma; UCEC: uterine corpus endometrial carcinoma; UCS: uterine carcinosarcoma; UVM: uveal melanoma; LumA: luminal A; LumB: luminal B; Her2: human epidermal growth factor receptor-2. **: *p* < 0.01; ***: *p* < 0.001.

The transcriptional levels of OXTR in some adjacent normal tissues and tumor tissues were analyzed using TIMER. The results showed that the expression of OXTR mRNA in COAD tissues was significantly higher than that in adjacent normal tissues (*P* < 0.001) (Figure 1a). Moreover, the KM curve analyzed in GEPIA2 indicated that in COAD, LGG, LUAD, MESO, SARC, and STAD (the squares marked by the red edge), patients with high levels of OXTR had poorer OS than those with low levels of OXTR (Figure 1b).

### 3.2 OXTR mRNA level is high in COAD patients from six datasets

**Figure 2:**
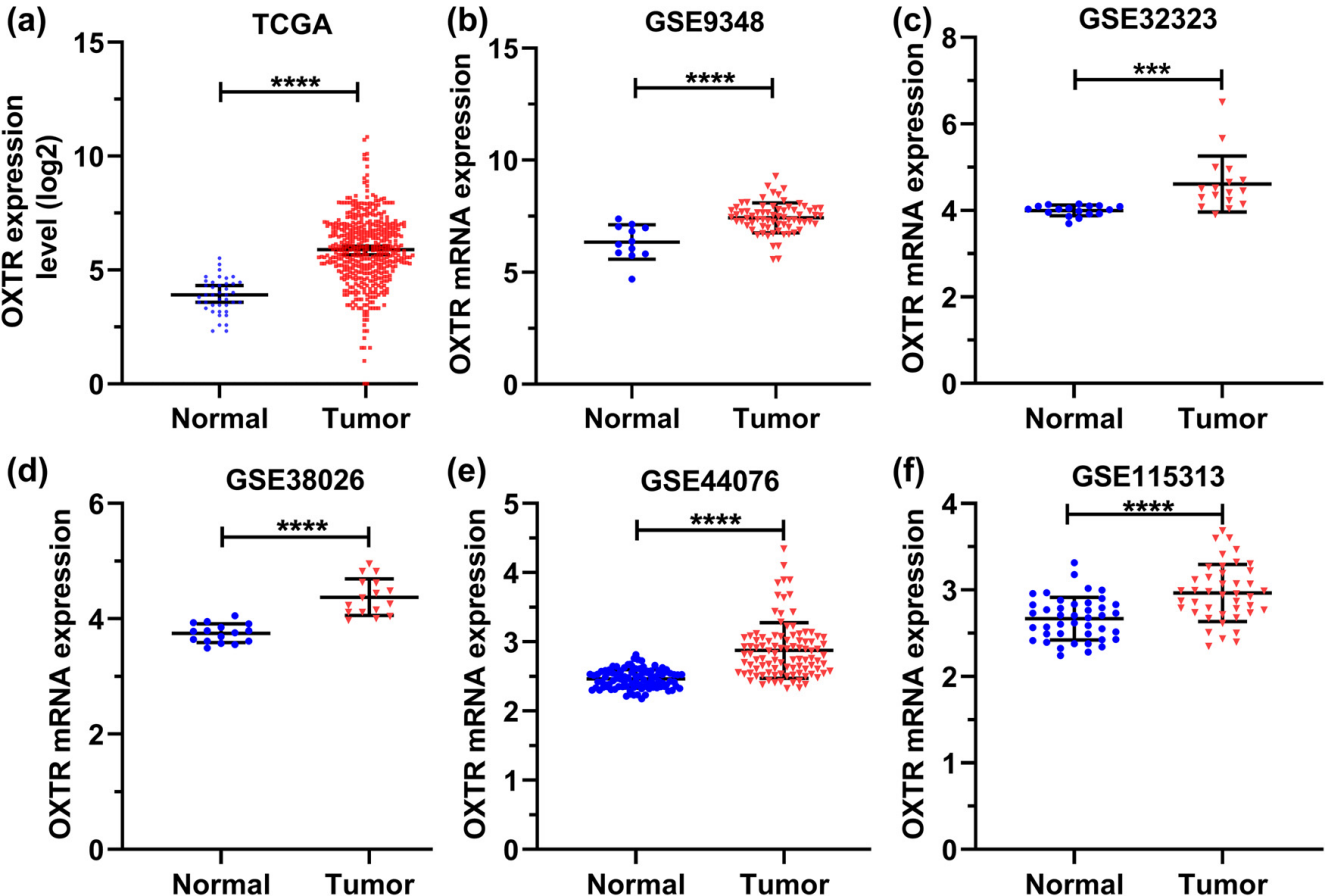
OXTR mRNA level is high in COAD patients from six datasets. (a–f) GraphPad Prism 8 was used to analyze the expression of OXTR in COAD samples from TCGA (a), GSE9348 (b), GSE32323 (c), GSE38026 (d), GSE44076 (e), and GSE115313 (f) databases. ***: *p* < 0.001; ****: *p* < 0.0001.

To further determine the expression pattern of OXTR in COAD tissues, the OXTR mRNA levels in COAD samples from TCGA, GSE9348, GSE32323, GSE38026, GSE44076, and GSE115313 database were analyzed. The result shows that OXTR transcription levels in COAD tissues are higher than that in normal tissues (Figure 2).

### 3.3 The level of OXTR is related to the progression of COAD

In order to investigate the role of upregulated OXTR mRNA in COAD patients, we further analyzed the relationship between OXTR mRNA levels and tumor progression. Patients (*n* = 428) were divided into groups with low or high OXTR expression using the survminer package in R language according to the survival data of COAD patients and the expression data of OXTR to investigate the clinical significance of OXTR expression. There were no significant differences between the two groups in gender, colon polyp history, hypermutation, tumor type, pathologic M, pathologic N, pathologic T, disease free survival (DFS), and progression-free interval (PFI) (all *P* > 0.05, Table 1). However, OXTR levels were strongly correlated with age, TNM stage, living status, and disease status (all *p* < 0.05, Table 1). Overall, these findings suggested that the level of OXTR is related to the progression of COAD.

**Table 1:** Association between OXTR expression and the clinical parameters in patients with COAD in TCGA

	OXTR expression			*p*-value
	Total	High	Low	
	(*N* = 428)	(*N* = 296)	(*N* = 132)	
Age (year)				**0.00566**
>60	296 (69.2%)	192 (64.9%)	104 (78.8%)	
≤60	132 (30.8%)	104 (35.1%)	28 (21.2%)	
Gender				1
Male	228 (53.3%)	158 (53.4%)	70 (53.0%)	
Female	200 (46.7%)	138 (46.6%)	62 (47.0%)	
TNM stage				**0.0309**
I	73 (17.1%)	43 (14.5%)	30 (22.7%)	
II	168 (39.3%)	119 (40.2%)	49 (37.1%)	
III	126 (29.4%)	84 (28.4%)	42 (31.8%)	
IV	61 (14.3%)	50 (16.9%)	11 (8.3%)	
History of colon polyps				0.0642
Yes	127 (29.7%)	78 (26.4%)	49 (37.1%)	
No	239 (55.8%)	171 (57.8%)	68 (51.5%)	
Unknown	62 (14.5%)	47 (15.9%)	15 (11.4%)	
Hypermutation				0.762
FALSE	111 (25.9%)	75 (25.3%)	36 (27.3%)	
Unknown	317 (74.1%)	221 (74.7%)	96 (72.7%)	
Type				0.81
Colon adenocarcinoma	363 (84.8%)	253 (85.5%)	110 (83.3%)	
Colon mucinous adenocarcinoma	60 (14.0%)	40 (13.5%)	20 (15.2%)	
Unknown	5 (1.2%)	3 (1.0%)	2 (1.5%)	
Pathologic M				0.102
M0	320 (74.8%)	212 (71.6%)	108 (81.8%)	
M1	61 (14.3%)	50 (16.9%)	11 (8.3%)	
MX	43 (10.0%)	31 (10.5%)	12 (9.1%)	
Unknown	4 (0.9%)	3 (1.0%)	1 (0.8%)	
Pathologic N				0.0909
N0	249 (58.2%)	169 (57.1%)	80 (60.6%)	
N1	102 (23.8%)	66 (22.3%)	36 (27.3%)	
N2	77 (18.0%)	61 (20.6%)	16 (12.1%)	
Pathologic T				0.0552
T1	10 (2.3%)	7 (2.4%)	3 (2.3%)	
T2	72 (16.8%)	42 (14.2%)	30 (22.7%)	
T3	296 (69.2%)	205 (69.3%)	91 (68.9%)	
T4	49 (11.4%)	41 (13.9%)	8 (6.1%)	
Unknown	1 (0.2%)	1 (0.3%)	0 (0%)	
Residual tumor				0.818
R0	314 (73.4%)	215 (72.6%)	99 (75.0%)	
R1–R2	25 (5.8%)	17 (5.7%)	8 (6.1%)	
Unknown	89 (20.8%)	64 (21.6%)	25 (18.9%)	
Living status				**0.0318**
Alive	334 (78.0%)	222 (75.0%)	112 (84.8%)	
Dead	94 (22.0%)	74 (25.0%)	20 (15.2%)	
Disease status				**0.0082**
No	159 (37.1%)	97 (32.8%)	62 (47.0%)	
Yes	22 (5.1%)	19 (6.4%)	3 (2.3%)	
Unknown	247 (57.7%)	180 (60.8%)	67 (50.8%)	
DFS				0.112
No	355 (82.9%)	238 (80.4%)	117 (88.6%)	
Yes	58 (13.6%)	46 (15.5%)	12 (9.1%)	
Unknown	15 (3.5%)	12 (4.1%)	3 (2.3%)	
PFI				0.0513
No	312 (72.9%)	207 (69.9%)	105 (79.5%)	
Yes	116 (27.1%)	89 (30.1%)	27 (20.5%)	

DFS: disease free survival; PFI: progression-free interval.

Bold values prompts *p* < 0.05.

### 3.4 The ROC curve shows that OXTR could distinguish COAD tissues from normal tissues

**Figure 3:**
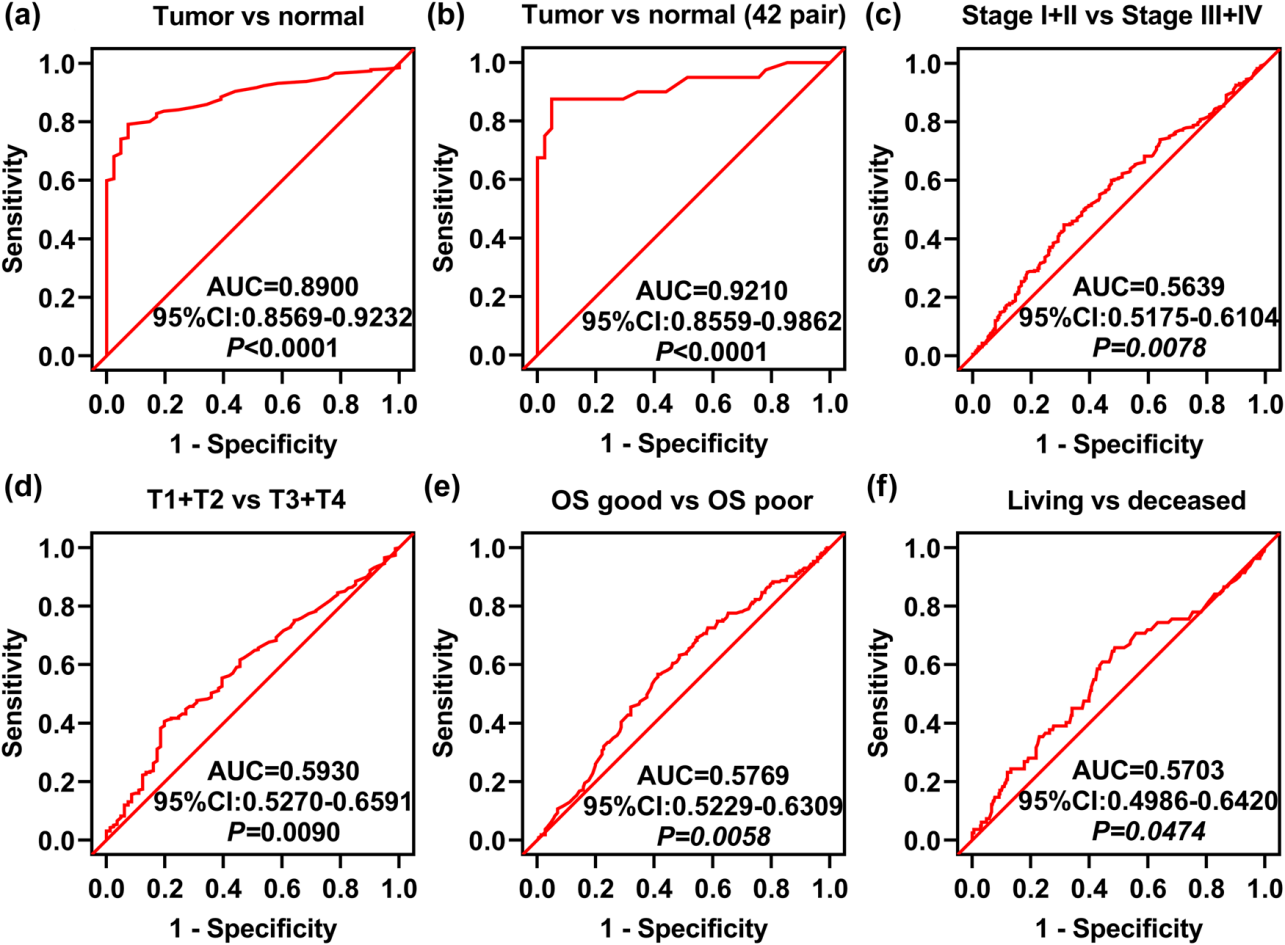
ROC curve analysis of OXTR expression related to the indicated clinical parameters in COAD patients. (a) The ROC curve analysis of COAD tissue and unpaired normal tissue. (b) The ROC curve analysis of COAD tissues and paired normal tissues. (c–f) ROC curve analysis of TNM stage (c), T stage (d), OS (e), and living status (f). AUC: area under the curve; OS: overall survival.

To confirm that OXTR mRNA level is associated with tumor progression and poor prognosis in patients, we utilized ROC curves. We found that OXTR could discriminate COAD tissues from normal tissues with an AUC of 0.8900 (95% CI: 0.8569–0.9232; *p* < 0.0001) (Figure 3a). Similarly, it also effectively distinguished COAD tissues from normal tissues in 42 paired samples with an AUC of 0.9210 (95% CI: 0.8559–0.9862; *p* < 0.0001) (Figure 3b). However, we found that OXTR cannot effectively distinguish COAD subgroups, including TNM stage (TNM stages I + II versus TNM stages III + IV, AUC = 0.5639, *p* = 0.0078, Figure 3c), T stage (stages T1 + T2 versus stages T3 + T4, AUC = 0.5930, *p* = 0.0090, Figure 3d), OS (OS-good versus OS-poor, AUC = 0.5769, *p* = 0.0058, Figure 3e), and living status (living versus deceased, AUC = 0.5703, *p* = 0.0474, Figure 3f). These results confirm that OXTR can be used as a biomarker of COAD, but it cannot effectively distinguish COAD tissues in different states.

### 3.5 COAD patients with high levels of OXTR are more likely to show short OS time

**Figure 4:**
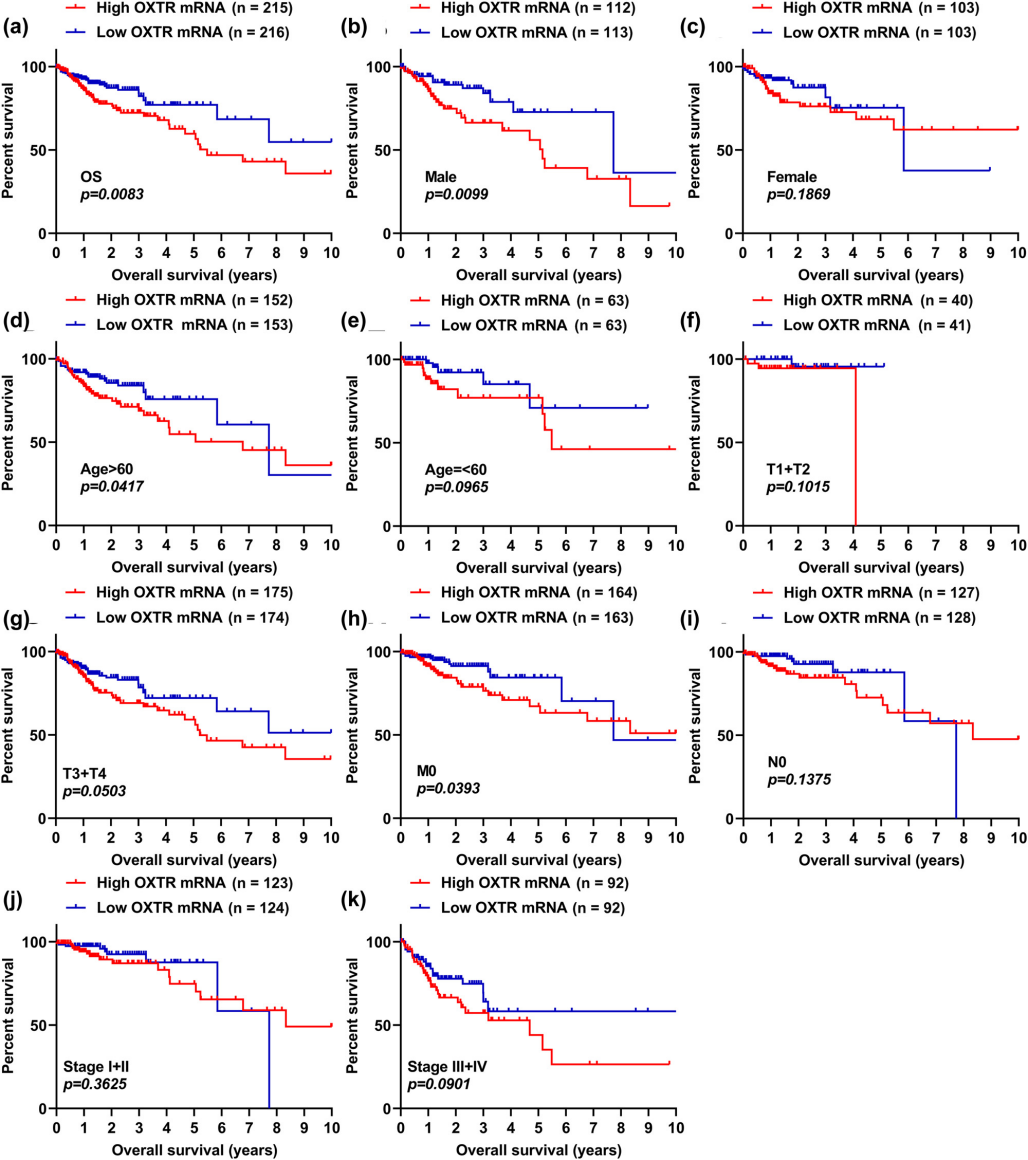
COAD patients with high levels of OXTR are more likely to show short OS time. (a) The correlation analysis between OXTR mRNA level and OS in COAD patients was performed by the KM curve. (b–k) KM curve analysis of male (b), female (c), age > 60 years (d), age ≤ 60 years (e), stages T1 + T2 (f), stages T3 + T4 (g), no metastasis (h), N0 stage (i), stages I + II (j), and stages III + IV (k) patients with COAD.

We used KM survival analysis to verify the relationship between OS time and OXTR mRNA level in COAD patients. Based on the median value of OXTR mRNA level in the TCGA data set, we divided 268 patients with COAD into high OXTR mRNA group and low OXTR mRNA group. Patients with high OXTR expression have shorter OS time than those with low OXTR expression (Figure 4a, *p =* 0.0083). Moreover, male (*p* = 0.0099) patients with high OXTR mRNA level showed shorter OS, and age > 60 years (*p* = 0.0417) and M0 stage (*p* = 0.0393) patients with high OXTR expression are more likely to exhibit a short OS time, but there was no significant correlation between OS time and OXTR level in females (*p* = 0.1869), age ≤ 60 years (*p* = 0.0965), T1 + T2 stage (*p* = 0.1015), T3 + T4 stage (*p* = 0.0503), N0 stage (*p =* 0.1375), stages I + II (*p* = 0.3625), and stages III + IV (*p* = 0.0901) patients (Figure 4b–k). All these data reveal that high levels of OXTR are associated with patients exhibiting a short OS time.

### 3.6 OXTR is involved in cell cycle regulation

**Figure 5:**
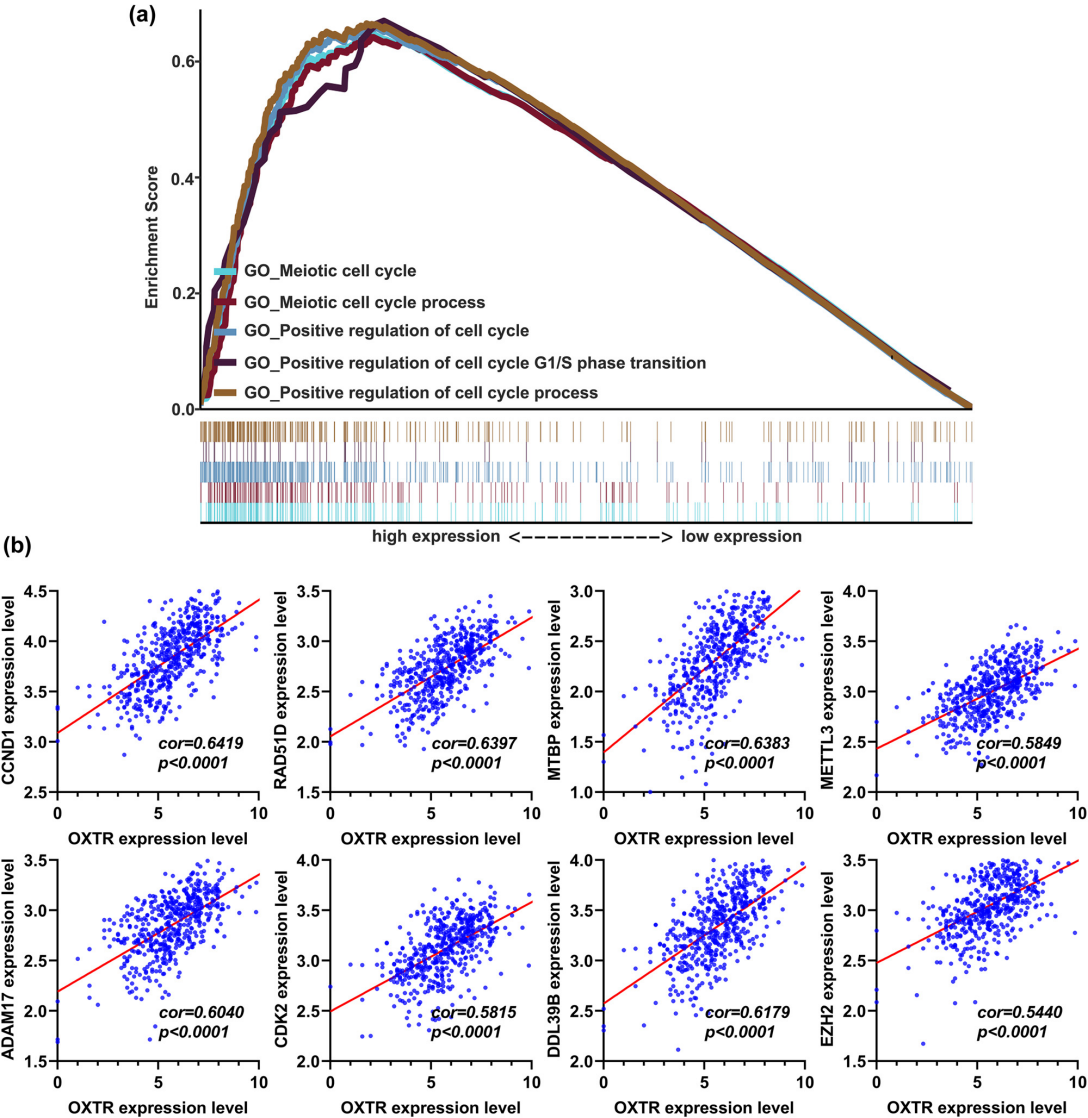
GO enrichment analysis of the correlation between OXTR mRNA level and genes related to cell cycle regulation. (a) Gene set enrichment analysis of the correlation between OXTR expression and mRNA levels of genes involved in cell cycle regulation. (b) Correlation analysis between OXTR expression and mRNA levels of genes involved in cell cycle regulation. GO, gene ontology.

To explore the association between OXTR expression and genes involved in cell cycle regulation, we performed GO enrichment analysis and found that high OXTR expression was associated with cell cycle regulation (Figure 5a). Furthermore, the expression of OXTR was strongly associated with genes involved in cell cycle regulation, including CCND1, RAD51D, MTBP, METTL3, ADAM17, CDK2, DDL39B, and EZH2 (Figure 5b). These findings show that high levels of OXTR are involved in COAD cell proliferation.

### 3.7 OXTR is involved in regulating four signaling pathways closely related to the occurrence and development of tumors

**Figure 6:**
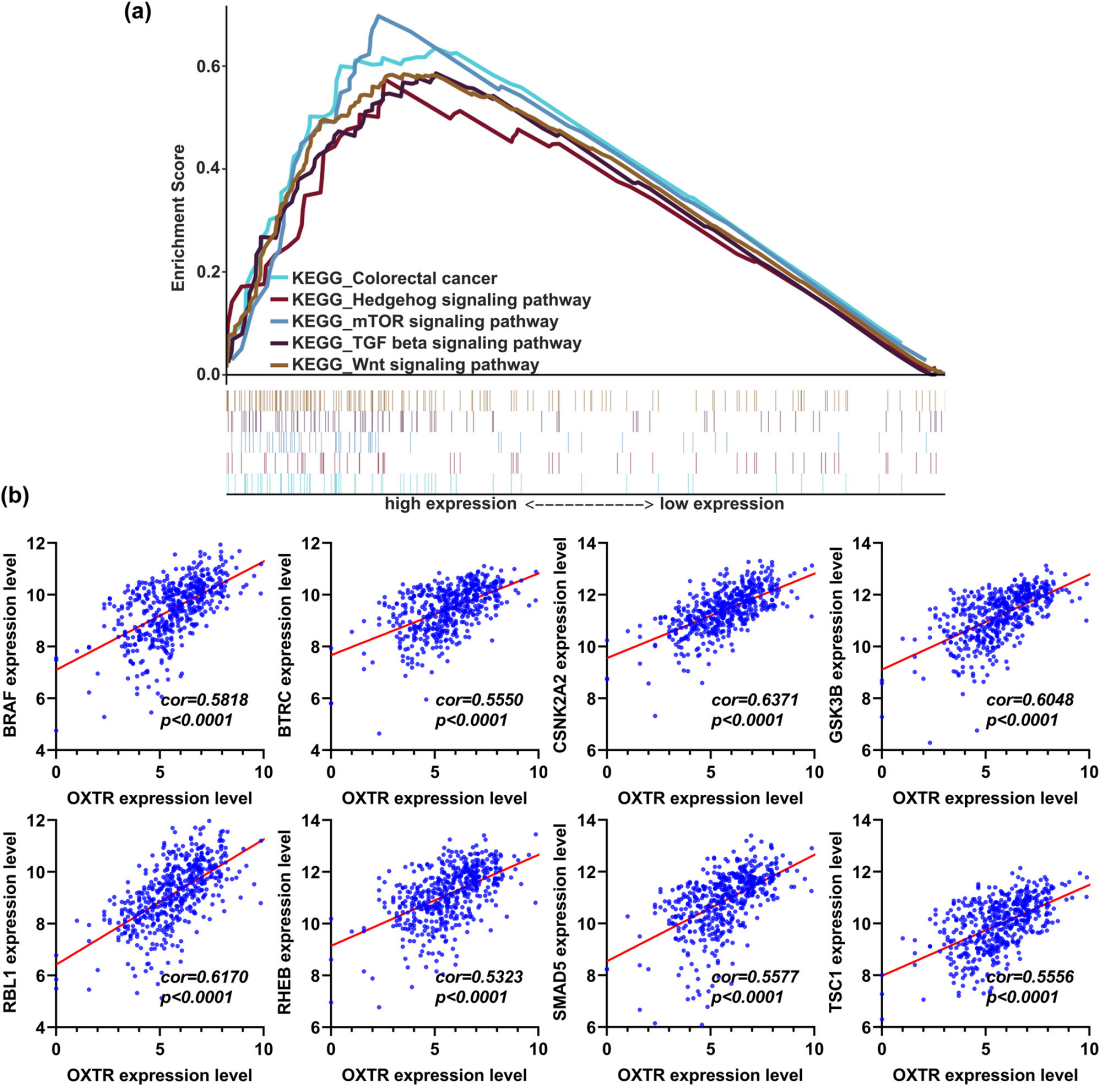
KEGG enrichment analysis of the correlation of OXTR mRNA level with colorectal cancer and four main signaling pathways in colorectal cancer. (a) Gene set enrichment analysis of the correlation between OXTR expression level and the expression of genes related to four signaling pathways in colorectal cancer. (b) Correlation analysis between the OXTR expression level and mRNA levels of genes related to four signaling pathways in COAD. KEGG: kyoto encyclopedia of genes and genomes.

We studied the relationship between OXTR levels and signaling pathways through KEGG enrichment analysis, finding that OXTR level was positively associated with four main well-studied signaling pathways in COAD, including the hedgehog, mTOR, TGF-β, and Wnt signaling pathways (Figure 6a). Moreover, OXTR level was positively associated with colorectal cancer (Figure 6a). In addition, OXTR levels were positively correlated with the levels of genes related to these pathways, including BRAF, BTRC, CSNK2A2, GSK3B, RBL1, RHEB, SMAD5, and TSC1 (Figure 6b).

### 3.8 PPI and gene co-expression network analysis

**Figure 7:**
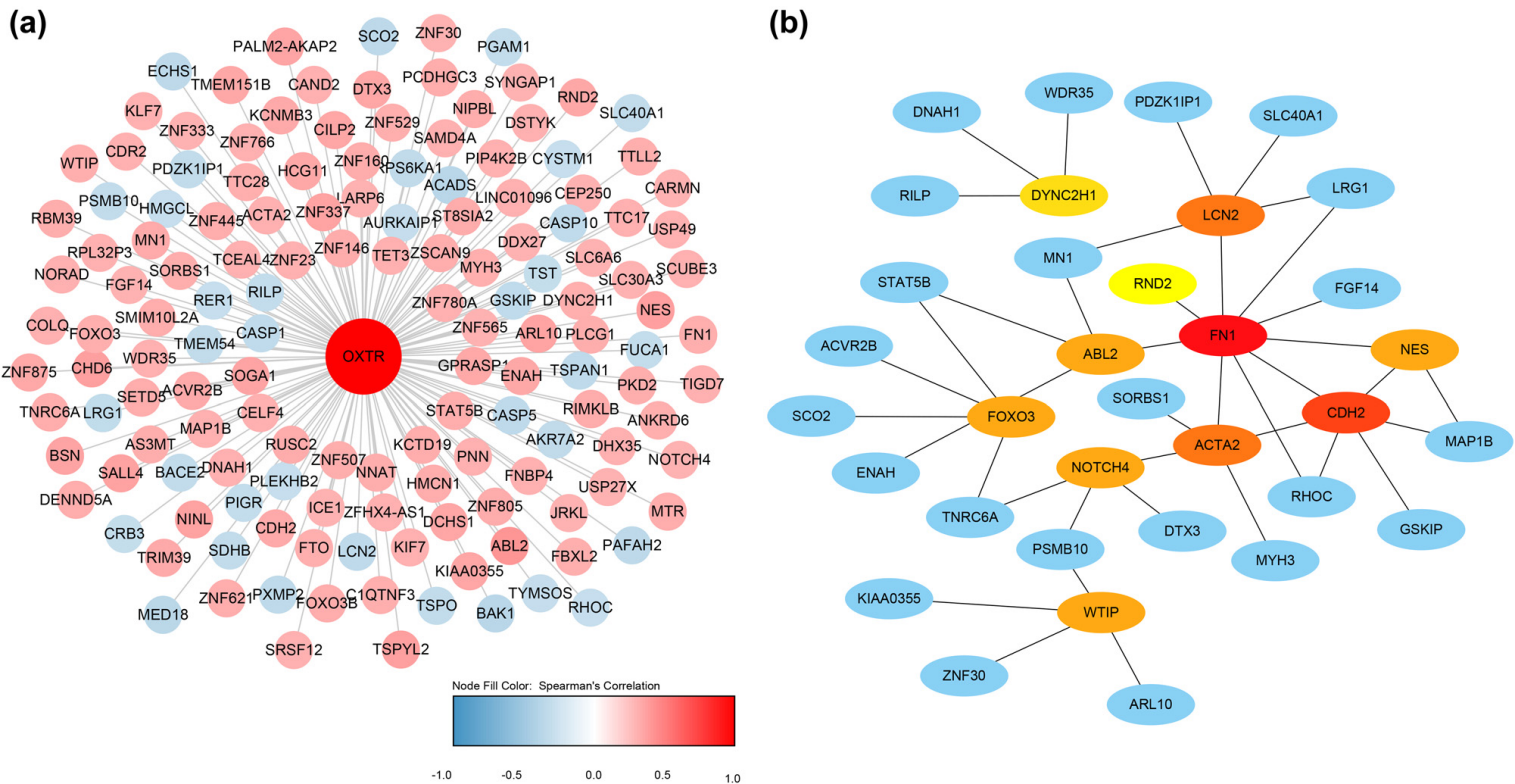
PPI and gene co-expression network analysis. (a) Hub genes of the PPI network. (b) Gene co-expression networks. Blue indicates genes that are negatively related to OXTR, and red indicates genes that are positively related to OXTR. The darker the color, the stronger the correlation.

Genes co-expressed with OXTR were screened using cBioPortal database. The Spearman correlation coefficient of a total of 146 genes was greater than 0.3 (Figure 7a). Subsequently, these genes were used for PPI network. After the removal of proteins that did not interact with other proteins, a total of 34 genes were found in the PPI network, including 10 nodes (DYNC2H1, LCN2, FN1, ABL2, FOXO3, NOTCH4, WTIP, ACTA2, CDH2, and NES) (Figure 7b). Unfortunately, no genes encoding proteins that interact with OXTR have been found (Figure 7b).

### 3.9 Correlations between OXTR expression and immune infiltration in COAD

**Figure 8:**
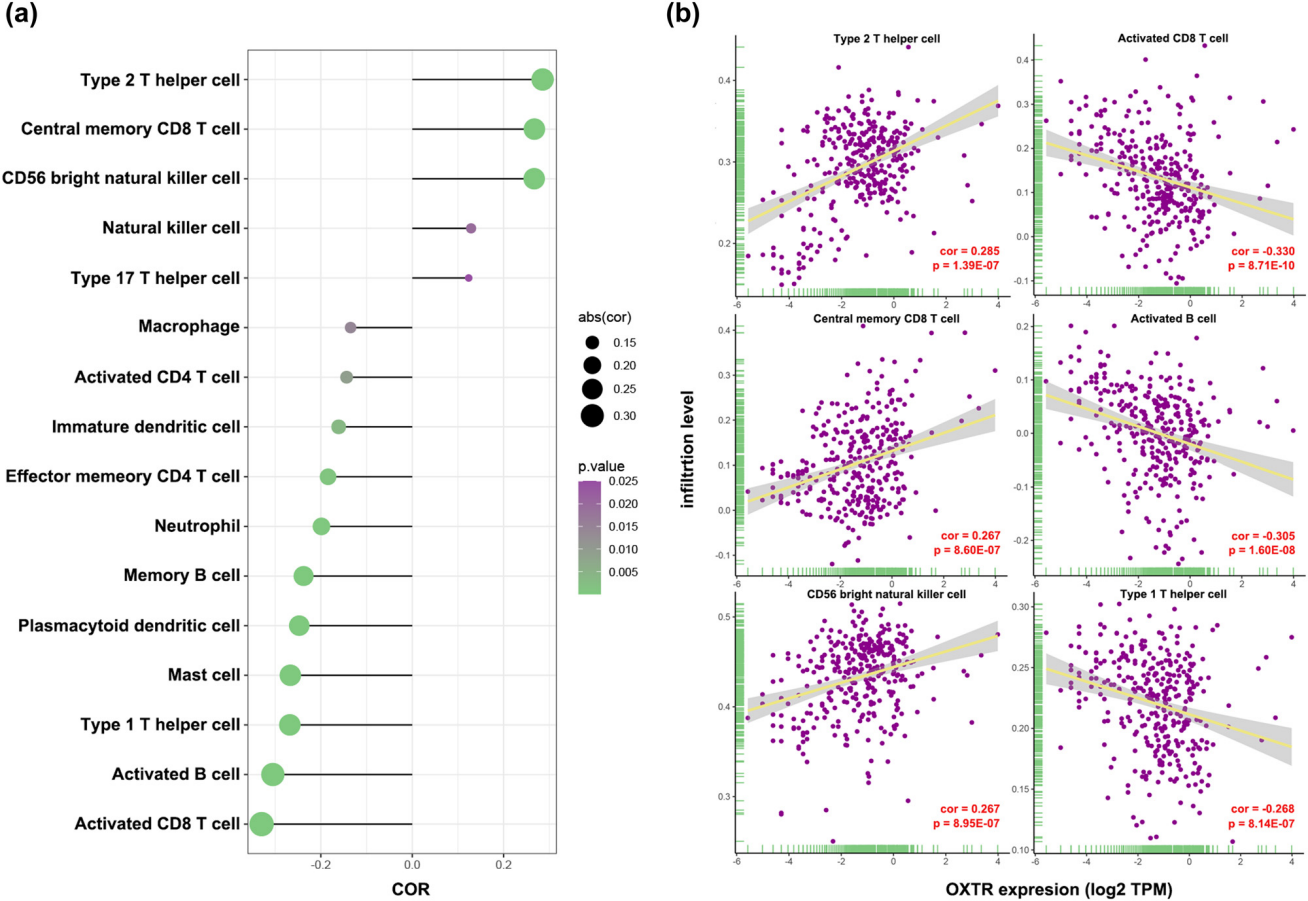
Correlations between OXTR expression and immune infiltration in COAD. (a) Rod diagram of the relationship between OXTR levels and infiltration levels of various immune cells. (b) Scatter plot of the association between OXTR levels and infiltration levels of type 2T helper cell, central memory CD8 T cell, CD57 bright natural killer cell, activated CD8 T cell, activated B cell, and Type 1T helper cell.

After analyzing the immune infiltration in COAD tissues, we found that OXTR level is positively correlated with the infiltration of type 2T helper cell, central memory CD8 T cell, and CD57 bright natural killer cell, while negatively correlated with the infiltration of activated CD8 T cell, activated B cell, and Type 1T helper cell (Figure 8).

### 3.10 Silencing OXTR inhibits cell proliferation, migration, and invasion

**Figure 9:**
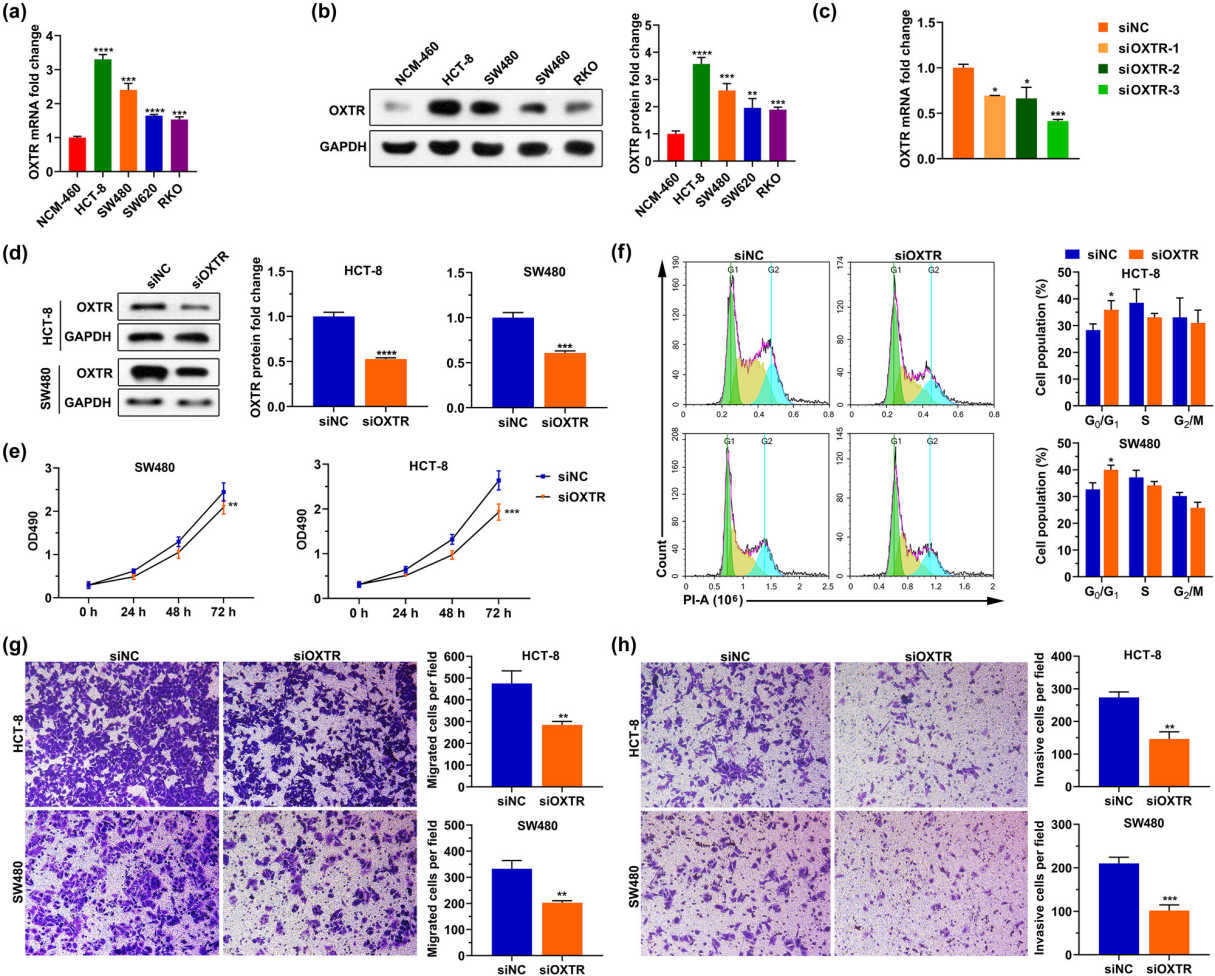
Silencing OXTR inhibits cell proliferation, migration, and invasion. (a and b) OXTR mRNA (a) and protein (b) levels in human normal colonic epithelial cell line (NCM-460) and COAD cell lines (HCT-8, SW480, SW620, and RKO). (c and d) After HCT-8 and SW480 cells were transfected with siOXTR, qPCR (c) and western blotting (d) assays were used to analyze the levels of OXTR mRNA and protein in the cells, respectively. (e‒h) After silencing OXTR, MTT assay, PI staining, and transwell assay were used to detect cell proliferation (e), cell cycle (f), migration (g), and invasion (h). siOXTR: small interfering RNA of OXTR; siNC: the negative control of siOXTR; PI: propidium iodide. *: *p* < 0.05, **: *p* < 0.01, ***: *p* < 0.001, ****: *p* < 0.0001.

In order to explore the effect of upregulated OXTR in COAD tissues on the biological processes of COAD cells, we silenced the expression of OXTR in HCT-8 and SW480 cells. We tested the expression of OXTR in human normal colon epithelial cell line (NCM-460) and COAD cell lines (HCT-8, SW480, SW620, and RKO), and found that the expression level of OXTR in COAD cell lines was higher than that in NCM-460 cells. Among them, the expression level of OXTR was the highest in HCT-8 cells, followed by that in SW480 cells (Figure 9a and b). Therefore, subsequent cell experiments were performed with HCT-8 and SW480 cells. We designed three siRNA of OXTR (siOXTR-1, siOXTR-2, and siOXTR-3), and their sequences are shown in Table 2. After they were transfected into cells for 48 h, qPCR was performed and it was found that siOXTR-3 had the most significant effect on reducing the OXTR mRNA level in cells (Figure 9c). Therefore, siOXTR-3 was used in subsequent experiments. In addition, transfection of siOXTR into cells also reduced the level of OXTR protein in the cells (Figure 9d). Moreover, decreasing OXTR inhibited cell proliferation, migration, and invasion, and arrested the cell cycle (Figure 9e–h). These findings indicate that the upregulated OXTR in COAD tissue may play a role in promoting cell proliferation, migration, and invasion.

**Table 2:** The sequences of siRNAs

Name	Sequence (5′-3′)
siNC	UUCUCCGAACGUGUCACGUTT
siOXTR-1	AUCUUGAAGCUGAUAAGGCCG
siOXTR-2	AGAUCUUGAAGCUGAUAAGGC
siOXTR-3	UGAUGAAAGUCAUCUUGACCG

siNC: the negative control of siRNA. siOXTR: the siRNA of OXTR.

## 4 Discussion

COAD is generally believed to be an illness derived from the accumulation of genetic and epigenetic mutations in epithelial cells. OXTR coupling with Gi and Gh proteins enables association with oxytocin to relay signals to trigger reproductive and social behavior [17,18]. Several previous studies have demonstrated that the activation of OXTR-mediated signaling promotes or prevents tumorigenesis and metastasis in multiple cancers, including breast cancer, non-small-cell lung cancer, prostate cancer, and ovarian cancer [20,21,28,29]. In the present study, we explored the role of OXTR in modulating progress and metastasis of COAD by bioinformatics analysis. Our results found that the mRNA OXTR upregulation was associated with growth and distant metastasis of COAD, and high mRNA level of OXTR indicated a poor prognosis in COAD patients. In addition, we also found that downregulating the expression of OXTR in COAD cells reduced the ability of cells to proliferate, migrate and invade, and blocked cell cycle progression.

Moreover, studies have shown that excessive activation of TGF-β, Wnt/β-catenin, Smad, Notch, MAPK, HIF-1, and mTOR signals in COAD led to the development of COAD [8,10,11,12]. GO and KEGG enrichment analyses also indicated that high OXTR expression was associated with loss of cell cycle regulation and significantly associated with four main signaling transduction pathways, including the hedgehog, mTOR, TGF-β, and Wnt signaling pathways. Therefore, we hypothesized that OXTR may be involved in overactivation of the mTOR, TGF-β, and Wnt signaling pathways to promote tumor progression, which needs further study. However, through PPI network analysis, no genes encoding proteins that interact with OXTR were found, indicating that further experimental studies are needed to understand the proteins interacting with OXTR.

This study lacks animal experiments and test results of OXTR protein in COAD tissue, so it is impossible to directly verify the influence of OXTR on COAD tumor growth, which is the limitation of this study. In addition, we found that OXTR levels did not have a significant effect on OS in females with COAD, but have a significant effect on OS in males with COAD. The reason why OXTR level had no significant effect on the OS of females with COAD may be that some female factors affected the effect of OXTR, or the sample size of females with COAD was not large enough, which still needs further investigation.

In conclusion, our findings clarified that the mRNA level of OXTR was elevated in COAD tissues and distant metastasis-prone COAD patients. Our results also suggest that COAD patients with high levels of OXTR have poorer OS than patients with low levels of OXTR. OXTR level was positively associated with hedgehog, mTOR, TGF-β, and Wnt signaling pathways, and regulation of cell cycle. OXTR expression was significantly correlated with the infiltration level of activated CD8 T cell and activated B cell. Moreover, knockdown of OXTR suppressed the proliferation, migration, and invasion of COAD cells, and blocked the cells in the G_0_/G_1_ phase. OXTR might be a potential therapeutic target for COAD.
